# A 48-Week, Randomized Controlled Trial of Doravirine for Individuals With HIV and Obesity on Integrase Inhibitors and Tenofovir Alafenamide: The Do IT Study (ACTG A5391)

**DOI:** 10.1093/cid/ciag196

**Published:** 2026-03-18

**Authors:** John R Koethe, Jordan E Lake, Amy Kantor, Laura Smeaton, Kristine Erlandson, Laura Moran, Pablo Belaunzaran-Zamudio, Alan Landay, Rafael E Campo, Paula Debroy, Jaclyn Ann Bennet, Jane O'Halloran, Win Min Han, Oladapo Alli, Michael Leonard, Roy M Gulick

**Affiliations:** Division of Infectious Diseases, Vanderbilt University Medical Center, Nashville, Tennessee, USA; Division of Infectious Diseases, University of Texas Health Science Center at Houston, Houston, Texas, USA; Center for Biostatistics in AIDS Research, Harvard T.H. Chan School of Public Health, Boston, Massachusetts, USA; Center for Biostatistics in AIDS Research, Harvard T.H. Chan School of Public Health, Boston, Massachusetts, USA; Division of Infectious Diseases, University of Colorado Anschutz Medical Campus, Aurora, Colorado, USA; ACTG Network Coordinating Center, Bethesda, Maryland, USA; Division of AIDS, National Institutes of Health, Bethesda, Maryland, USA; Departments of Internal Medicine and Microbiology & Immunology, University of Texas Medical Branch at Galveston, Galveston, Texas, USA; Global Medical and Scientific Affairs, Merck Research Laboratories, Merck & Co., Inc., Rahway, New Jersey, USA; Division of Infectious Diseases, University of Texas Health Science Center at Houston, Houston, Texas, USA; Clinical HIV Research Unit, Internal Medicine Department, School of Clinical Medicine, Faculty of Health Sciences, University of the Witwatersrand, Johannesburg, South Africa; Center for Experimental Pathogen Host Research (CEPHR), University College Dublin, Dublin, Ireland; HIV-NAT, Thai Red Cross AIDS and Infectious Disease Research Centre, Bangkok, Thailand; Division of AIDS, National Institutes of Health, Bethesda, Maryland, USA; Division of Infectious Diseases, Vanderbilt University Medical Center, Nashville, Tennessee, USA; Division of Infectious Diseases, Weill Cornell Medicine, New York, New York, USA

**Keywords:** HIV, weight, doravirine, tenofovir alafenamide, obesity

## Abstract

**Background:**

Integrase inhibitors (INSTI) and tenofovir alafenamide (TAF) have been associated with greater weight gain compared to nonnucleoside reverse transcriptase inhibitors (NNRTIs) and tenofovir disoproxil fumarate (TDF), but the effects of an antiretroviral regimen switch on weight are unclear.

**Methods:**

This 48-week, 3–parallel group, open-label, multicenter, randomized controlled trial (NCT04636437) in people with HIV and obesity on an INSTI (bictegravir, dolutegravir, or raltegravir) with TAF/emtricitabine (FTC) assessed whether switching to the NNRTI doravirine (DOR) with TAF/FTC or TDF/FTC results in weight loss or stabilization. Treatment effects were estimated using linear regression adjusted for sex, race, and entry weight.

**Results:**

Of the 147 participants randomized, 145 initiated their assigned treatment. At entry, median age was 49 years; body mass index was 34.9 kg/m^2^, and time on INSTI + TAF/FTC was 3.4 years; 49% were female and 53% Black. After 48 weeks, the estimated mean change in weight was −0.47% (95% CI: −2.09, 1.14) for the DOR + TAF/FTC arm, −2.73% (−4.22, −1.23) for DOR + TDF/FTC, and −1.84% (−3.37, −0.30) for INSTI + TAF/FTC. The estimated mean difference in weight change at 48 weeks for DOR versus INSTI (both with TAF/FTC) was 1.36 percentage points (97.5% CI: −1.20, 3.92) and −0.89 percentage points (−3.34, 1.57) for DOR + TDF/FTC versus INSTI + TAF/FTC. There was no evidence of variation by sex or race, nor of treatment differences for changes in fasting lipids, insulin resistance, fat mass, or bone mineral density.

**Conclusions:**

In people with HIV and obesity, switching from an INSTI + TAF/FTC regimen to DOR/FTC with either TAF or TDF did not produce clinically meaningful differences in weight change or metabolic health after 48 weeks.

## Teaser

The prospective, randomized, multicenter A5391 trial found that switching from INSTI- and TAF-containing antiretroviral therapy to an NNRTI, with or without TDF, does not reduce weight or improve metabolic outcomes in people with HIV who already have obesity.

**(See the Editorial Commentary by Martínez on pages e90–1.)**

A growing proportion of people with HIV-1 (PWH) across diverse geographic areas and demographics are overweight or obese [1, 2]. Above “normal” weight from any cause increases the risk of comorbid conditions, including diabetes, steatotic liver disease, and cardiovascular disease [3–7]. Several recent studies observed greater weight gain among treatment-naive PWH starting integrase strand transfer inhibitor (INSTI)–based regimens compared to nonnucleoside reverse transcriptase (NNRTI)–based regimens [8, 9], as well as those receiving tenofovir alafenamide (TAF) compared to tenofovir disoproxil fumarate (TDF) [9, 10]. Taken together, these findings have been interpreted as evidence for a weight-promoting effect of newer INSTI- and TAF-containing regimens as compared with older antiretroviral therapy (ART) regimens.

The variability in weight gain early after treatment initiation by medication class and agent raises the possibility that ART changes, specifically a switch off of an INSTI or TAF, might produce weight stabilization or weight loss and metabolic health improvement, particularly among women, Black individuals, and other subgroups at highest risk of excessive (>10%) weight gain and obesity [10]. Less weight gain with NNRTIs has raised interest in this class for individuals with obesity or excessive weight gain after treatment initiation. Doravirine (DOR), a newer NNRTI, was associated with modest weight gain in treatment-naive clinical trials [11], as well as weight loss in small observational switch studies [12, 13]. Additionally, harnessing the known weight-suppressive properties of TDF could offer therapeutic benefit, but this is balanced against the potential for reductions in bone density and renal function [14]. The ACTG A5391 “Do-IT” trial was developed to assess whether a switch from an INSTI- to a DOR-containing regimen, as well as from a TAF- to a TDF-containing regimen, would reduce weight and improve metabolic health among adults with HIV and obesity.

## METHODS

A5391 was a 48-week, 3–parallel group, open-label, multicenter (25 US sites), randomized controlled trial in PWH with obesity (a body mass index [BMI] ≥30 kg/m^2^) on an INSTI and TAF + emtricitabine (FTC) to assess whether a switch to either DOR with TAF/FTC or DOR with TDF/FTC results in weight change compared to continued INSTI-based ART. The study was sponsored by the Division of AIDS (DAIDS) of the National Institute of Allergy and Infectious Diseases (NIAID; part of the National Institutes of Health). Eligibility requirements included age ≥18 years and receipt of an ART regimen containing bictegravir (BIC), dolutegravir (DTG), or raltegravir (RAL) with TAF/FTC for at least 48 weeks and virologic suppression (HIV-1 RNA <50 copies/mL or below the local lower limit of HIV-1 RNA detection). Elvitegravir use was excluded due to the potential confounding effects of cobicistat.

A protocol revision in August 2022 changed the initial eligibility criteria from a BMI ≥27.5 kg/m^2^ and historical evidence of a >10% unintentional weight gain in the first 1–3 years on an INSTI + TAF/FTC regimen to a BMI ≥30 kg/m^2^ alone due to limited historical weight record availability at some sites, particularly during the SARS-CoV-2 pandemic (weight history was recorded on all participants, as available). Exclusion criteria included historical or current evidence of major reverse transcriptase mutations associated with NNRTI resistance or the K65R/E/N or M184V/I mutations [15], as well as a history of prior virologic failure. Individuals with any history of significant renal toxicity while taking TDF or a previous diagnosis of osteoporosis or osteopenia were excluded. Individuals were also excluded if they had an anticipated start or cessation of any medication known to significantly alter body weight, or intention to initiate a weight loss program.

Eligible participants were randomized (1:1:1) to 1 of 3 study arms (switch to standard-dose, once-daily DOR [100 mg] + TAF/FTC [25 mg/200 mg] or DOR [100 mg] + TDF/FTC [300 mg/200 mg], or continue their current INSTI + TAF/FTC regimen) using centrally computer-generated, permuted blocks with institutional balancing and stratification by both sex and Black versus non-Black race to conceal allocation from participants and the study team until study entry. Given the risk for greater weight gain previously observed among women and Black individuals receiving INSTI-containing regimens [8, 10], prespecified enrollment targets of >50% women (defined as female sex at birth) and >50% Black participants were set, which required placing sex enrollment limits after study initiation. Doravirine was provided by the manufacturer (Merck & Co., Inc., Rahway, NJ, United States), and NRTIs and INSTIs were obtained by participants through routine clinical care. Study visits were at weeks 0 (entry), 4, 12, 24, 36, and 48 and included standardized measurements of body weight and waist circumference as well as safety assessments (graded per the DAIDS severity scale) [16]. Triglycerides, high-density lipoprotein (HDL), glucose, and insulin (measured by duplicate radioimmunoassay) were measured after a minimum 8 hour fast at weeks 0, 24 and 48; low-density lipoprotein (LDL) was calculated using the Martin–Hopkins equation, and a homeostatic model assessment of insulin resistance (HOMA-IR) score was calculated as (glucose [mg/dL] × insulin [mU/L])/405. Insulin and HIV-1 RNA measurements were centralized, while other laboratory values were measured using standardized assays in CLIA-certified laboratories approved by DAIDS. Hip and lumbar (L1-L4) spine bone mineral densities (BMD) and total and regional lean and fat mass were measured at weeks 0 and 48 by whole-body dual-energy X-ray absorptiometry (DEXA) scan. A food security score was calculated at entry using the USDA Adult Food Security Questionnaire short form, and a physical activity score using the International Physical Activity Questionnaire short form.

### Statistical Considerations

The original sample size of 222 was based on 80% power to detect a treatment effect of 5 percentage points of weight change at week 48 between each switch arm and the control group, assuming a standard deviation of weight change of 9% and a 2.5% significance level, to reflect 2 pairwise comparisons for the primary trial objective and 10% sample size inflation for loss to follow-up. The selection of a 5 percentage point difference in weight change as a clinically meaningful difference was based on studies showing reductions in diabetes risk and intrahepatic triglyceride content, and improved insulin sensitivity, with this degree of weight loss [17]. Following a planned interim analysis in June 2023 by the independent study monitoring committee, the sample size was adjusted to enable completion of the trial given slower-than-expected recruitment. Blinded sample size reestimation, using an estimate of the standard deviation on the primary outcome of weight change over all arms of 5.3%, led to 98% statistical power for the initially hypothesized effect size of 5 percentage points with only 150 participants (45 per arm after adjustment for 10% loss to follow-up). This reduced sample size had 80% statistical power to detect a 3.5 percentage point effect size. Projections under a more conservative standard deviation of 7% (80% power for a 4.6 percentage point effect size) provided confidence that reducing the sample size would not jeopardize power for the primary objective.

The primary outcome was analyzed among participants who received ≥1 dose of study treatment and who were assessed for weight at week 48. The mean change from entry (randomization) to 48 weeks, along with the associated 95% CIs, was estimated for each treatment arm using a linear regression model that adjusted for both stratification factors (sex and Black vs non-Black race) and weight at entry. Treatment comparisons between each investigational arm (DOR + TAF/FTC and DOR + TDF/FTC) and the control arm (INSTI + TAF/FTC) were summarized by mean estimated difference and least squares-based 97.5% CI (2.5% significance-level adjustment based on 2 pairwise comparisons for the primary objective). The primary analysis analyzed participants as randomized. Prespecified secondary analyses were conducted among the subset with a documented >10% weight gain on an INSTI prior to trial participation and among those without on-study use of concomitant medications that could potentially affect weight. Tests for interaction between primary outcome treatment effect and prespecified subgroups were performed for sex, gender (cis-female or trans-female on hormones at entry vs other), race (Black vs non-Black), race/ethnicity (Black, non-Hispanic vs Hispanic [any race] vs non-Black, non-Hispanic), age (<50 vs ≥50 years), entry INSTI (DTG vs BIC), and history of a >10% weight gain on an INSTI-based regimen (yes, no, unknown). To evaluate the impact of missing weight measurements at week 48, prespecified sensitivity analyses using multiple imputation were performed, assuming both missing at random and missing not at random. A fully conditional imputation algorithm was used, and the model included both stratification factors (sex at birth and race), treatment, and all observed weight measurements for each participant. A similar analysis approach was used to assess treatment differences in the change from baseline for other outcomes. The serum lipid analyses excluded individuals receiving lipid-lowering medications, and the HOMA-IR analysis excluded those receiving insulin (but not stable doses of oral antidiabetic agents). All statistical analyses were performed using SAS for Linux.

Members of the community, including individuals with HIV, provided input throughout protocol development. The results were presented at an annual ACTG meeting attended by community members, and the ensuing discussion informed the interpretation and conclusions drawn from the study results. The study protocol was reviewed and approved by the ACTG single Institutional Review Board (IRB) and the IRBs at the clinical trials sites, and all participants provided written informed consent. An ACTG network–appointed, independent study monitoring committee reviewed trial design, conduct, and safety information (but not efficacy, as per the trial's monitoring plan) by blinded treatment arm yearly. The study is registered on ClinicalTrials.gov (NCT04636437).

## RESULTS

Between July 2021 and November 2023, 147 participants were randomized, and 145 initiated study treatment (DOR + TAF/FTC n = 47, DOR + TDF/FTC n = 49, INSTI + TAF/FTC n = 49). At entry, median age was 49 years; BMI 34.9 kg/m^2^; waist circumference 111 cm, CD4^+^ T-cell count 688 cells/mm^3^, and 49% female, 53% Black, and 18% Hispanic/Latino (Table 1). The median time on INSTI + TAF/FTC was 3.4 years. Before entry, 86% were taking BIC, 13% DTG, and 1% RAL, all with TAF/FTC. Sixty-five percent of participants had an unintentional ≥10% weight gain in the first 1–3 years of INSTI-based ART (29% with <10% weight gain or known medical reason for weight change ≥10%, 6% without available data). Entry BMI values were similar before and after the protocol change in the minimum eligible level (median 34.6 and 35.3 kg/m^2^, respectively); however, 8 participants had a BMI <30 kg/m^2^ at entry, with the lowest being 28.6 kg/m^2^.

**Table 1.** Demographics and Clinical and Metabolic Characteristics at Study Entry

		Overall (N = 145)	DOR + TAF/FTC (N = 47)	DOR + TDF/FTC (N = 49)	INSTI + TAF/FTC (N = 49)
Age (y)	Median (Q1, Q3)	49 (40, 57)	48 (36, 57)	50 (39, 55)	52 (42, 58)
	50+	72 (50%)	19 (40%)	25 (51%)	28 (57%)
Sex at birth	Female	71 (49%)	23 (49%)	24 (49%)	24 (49%)
Gender identity^a^	Cis-female	71 (49%)	23 (49%)	24 (49%)	24 (49%)
	Cis-male	69 (48%)	23 (49%)	24 (49%)	22 (45%)
	Gender queer	2 (1%)	1 (2%)	0 (0%)	1 (2%)
	Transgender female	3 (2%)	0 (0%)	1 (2%)	2 (4%)
Race^a^	White	62 (43%)	22 (47%)	21 (43%)	19 (39%)
	Black or African American	77 (53%)	24 (51%)	26 (53%)	27 (55%)
	Other/unknown	6 (4%)	1 (2%)	2 (4%)	3 (6%)
Ethnicity^a^	Hispanic or Latino	26 (18%)	10 (21%)	11 (22%)	5 (10%)
	Not Hispanic or Latino	117 (81%)	36 (77%)	38 (78%)	43 (88%)
	Unknown	2 (1%)	1 (2%)	0 (0%)	1 (2%)
CD4 (cells/mm^3^)	Median (Q1, Q3)	688 (504, 959)	635 (403, 959)	688 (529, 1071)	720 (556, 910)
Nadir CD4	<50	23 (16%)	9 (19%)	9 (19%)	5 (10%)
	50 to <200	30 (21%)	14 (30%)	6 (13%)	10 (20%)
	200+	91 (63%)	24 (51%)	33 (69%)	34 (69%)
History of AIDS	Yes	55 (38%)	23 (49%)	16 (33%)	16 (33%)
HIV-1 RNA (copies/mL)	<20	5 (3%)	0 (0%)	2 (4%)	3 (6%)
	<40	135 (93%)	44 (94%)	46 (94%)	45 (92%)
	100 to <400	3 (2%)	1 (2%)	1 (2%)	1 (2%)
Screening ART regimen	Bictegravir, TAF/FTC	125 (86%)	39 (83%)	42 (86%)	44 (90%)
	Dolutegravir, TAF/FTC	19 (13%)	8 (17%)	6 (12%)	5 (10%)
	Raltegravir, TAF/FTC	1 (<1%)	0 (0%)	1 (2%)	0 (0%)
Years on baseline ART regimen	Median (Q1, Q3)	3.41 (2.43, 4.27)	3.38 (1.74, 4.25)	3.48 (2.61, 4.47)	3.36 (2.49, 4.27)
Weight (kg)	Median (Q1, Q3)	99.7 (91.2, 117.8)	107.3 (90.4, 124.8)	95.9 (89.7, 120.7)	101.3 (92.7, 113.0)
BMI (kg/m^2^)	Median (Q1, Q3)	34.9 (32.3, 39.3)	36.9 (33.2, 41.3)	34.4 (31.7, 39.3)	34.9 (32.3, 39.2)
	25–29.9	8 (6%)	4 (9%)	1 (2%)	3 (6%)
	30–34.9	65 (45%)	16 (34%)	27 (55%)	22 (45%)
	35–39.9	39 (27%)	15 (32%)	11 (22%)	13 (27%)
	40+	33 (23%)	12 (26%)	10 (20%)	11 (22%)
Waist circumference (cm)	Median (Q1, Q3)	111.1 (101.8, 119.0)	112.4 (100.8, 120.7)	109.5 (104.0, 117.9)	111.0 (99.7, 118.5)
Historical weight gain^b^	Yes	94 (65%)	33 (70%)	34 (69%)	27 (55%)
	No	42 (29%)	12 (26%)	11 (22%)	19 (39%)
	Unknown	9 (6%)	2 (4%)	4 (8%)	3 (6%)
Hemoglobin A1C (%)	Median (Q1, Q3)	5.60 (5.40, 6.00)	5.60 (5.30, 6.00)	5.60 (5.40, 5.90)	5.70 (5.40, 6.00)
Glucose (mg/dL)	Median (Q1, Q3)	95.0 (87.0, 106)	96.0 (88.0, 111)	93.0 (88.0, 102)	96.0 (86.0, 111)
Insulin (μU/mL)	Median (Q1, Q3)	23.8 (15.6, 37.6)	28.8 (17.2, 39.8)	20.0 (15.4, 32.8)	22.7 (13.8, 37.6)
HOMA-IR	Median (Q1, Q3)	6.16 (3.35, 9.52)	6.60 (3.89, 10.6)	4.58 (3.27, 8.78)	6.30 (2.97, 8.96)
Triglycerides (mg/dL)	Median (Q1, Q3)	103 (75.0, 148)	93.0 (77.0, 149)	109 (80.0, 137)	100 (63.0, 165)
LDL cholesterol (mg/dL)	Median (Q1, Q3)	103 (78.6, 126)	104 (89.0, 132)	112 (91.0, 125)	91.0 (71.0, 117)
HDL cholesterol (mg/dL)	Median (Q1, Q3)	47.0 (39.0, 56.0)	45.0 (38.0, 53.0)	47.9 (40.0, 56.0)	50.0 (42.0, 59.0)
Creatinine clearance (mL/min)	Median (Q1, Q3)	84.3 (73.0, 102)	90.0 (71.0, 104)	82.9 (77.5, 100)	81.3 (73.0, 98.3)
Food security^a^	High food security	88 (61%)	30 (65%)	34 (69%)	24 (49%)
	Marginal food security	27 (19%)	9 (20%)	8 (16%)	10 (20%)
	Low food security	17 (12%)	5 (11%)	4 (8%)	8 (16%)
	Very low food security	12 (8%)	2 (4%)	3 (6%)	7 (14%)
	Missing	1	1	0	0
Physical activity^a^	High	53 (41%)	13 (34%)	21 (45%)	19 (42%)
	Moderate	39 (30%)	9 (24%)	15 (32%)	15 (33%)
	Low	38 (29%)	16 (42%)	11 (23%)	11 (24%)
	Missing	15	9	2	4

Abbreviations: AIDS, acquired immune deficiency syndrome; ART, antiretroviral therapy; BMI, body mass index; HDL, high-density lipoprotein; HOMA-IR, homeostatic model assessment of insulin resistance; LDL, low-density lipoprotein.

^a^Collected by self-report.

^b^Historical weight gain was defined as unintentional >10% weight gain in the 1–3 years after initiating or switching to INSTI-based ART, with no other medically apparent reason to readily explain the weight gain.

Among the 145 participants who initiated study treatment as assigned and comprised the safety analysis population, 127 completed 48 weeks and comprised the primary efficacy analysis population (DOR + TAF/FTC n = 39, DOR + TDF/FTC n = 45, INSTI + TAF/FTC n = 43; see Supplementary Figure 1 for early discontinuation reasons). Overall, self-reported, 4-day recall of adherence to study medications was 93% (128/138) at week 24 and 94% (119/127) at week 48. The number of participants who started or changed a medication with potentially significant effects on weight was similar across arms (DOR + TAF/FTC 33% [15/46], DOR + TDF/FTC 29% [14/48], INSTI + TAF/FTC 33% [16/49]) (Supplementary Table 1). Thirty-eight percent of participants across all arms were using lipid-lowering medications (DOR + TAF/FTC 30% [14/46], DOR + TDF/FTC 40% [19/48], INSTI + TAF/FTC 45% [22/49]).

### Primary Outcome of Weight Change at 48 Weeks

**Figure 1.**
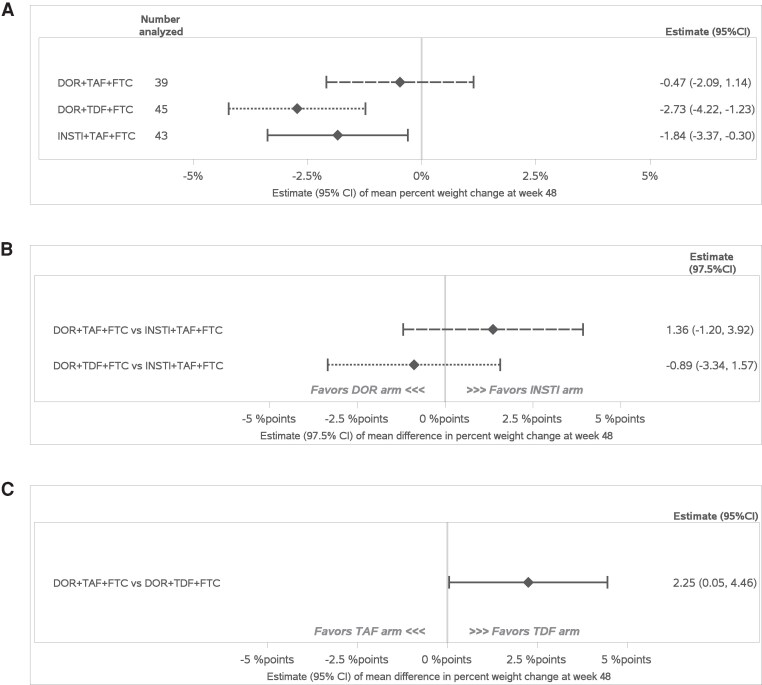
*A*, Estimated mean change in weight at 48 weeks by study arm. *B*, Estimated mean treatment differences for DOR + TAF/FTC and DOR + TDF/FTC versus INSTI + TAF/FTC at 48 weeks. *C*, Estimated treatment differences for DOR + TAF/FTC versus DOR + TDF/FTC at 48 weeks.

More than 50% of participants in all 3 study arms lost weight over 48 weeks (observed average: DOR + TAF/FTC −0.8 kg, DOR + TDF/FTC −2.9 kg, INSTI + TAF/FTC arm −1.8 kg). The estimated mean change (95% CI) in weight at 48 weeks was −0.47% (−2.09, 1.14) for DOR + TAF/FTC, −2.73% (−4.22, −1.23) for DOR + TDF/FTC, and −1.84% (−3.37, −0.30) for INSTI + TAF/FTC arm (Figure 1*A*). The estimated mean treatment difference (97.5% CI) was 1.36 percentage points (−1.20, 3.92) for DOR + TAF/FTC versus INSTI + TAF/FTC and −0.89 percentage points (−3.34, 1.57) for DOR + TDF/FTC versus INSTI + TAF/FTC (Figure 1*B*). When comparing the DOR arms, the mean treatment difference (95% CI) for DOR + TAF/FTC versus DOR + TDF/FTC was 2.25 percentage points (0.05, 4.46), favoring the TDF-containing arm (Figure 1*C*). However, all CI limits for the treatment difference in weight change were within 5 percentage points. There were no subgroup treatment differences between the DOR-containing ART versus INSTI + TAF/FTC (sex at birth, gender, race, race/ethnicity, age, entry INSTI, historical ART-related weight gain; Supplementary Figure 2). Estimated mean treatment differences for the change in waist circumference were not significant (Supplementary Figure 3). Sensitivity analyses using multiple imputation yielded similar results, as did analyses limited to those without use of concomitant medications that could potentially affect weight on study or history of a >10% weight gain in the first 3 years on an INSTI-based regimen.

### Fasting Lipids and Insulin Resistance Change at 48 Weeks

**Figure 2.**
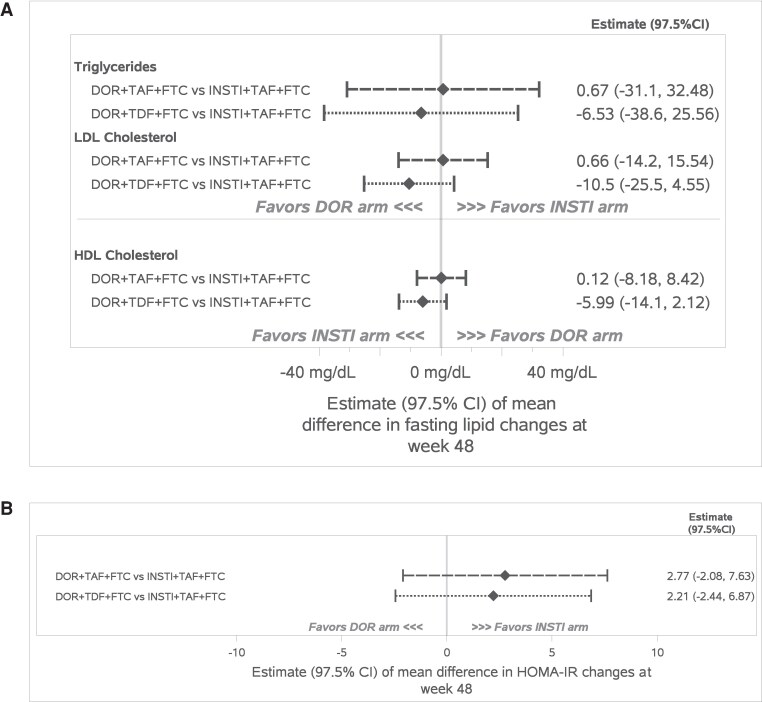
*A*, Estimated mean treatment differences in changes in fasting lipids and (*B*) homeostatic model assessment of insulin resistance (HOMA-IR) at 48 weeks. Fasting lipid analyses excluded participants on statins, and the HOMA-IR analysis excluded participants on insulin.

The estimated mean changes in triglycerides at 48 weeks were 5.0 mg/dL (n = 26; 95% CI: −13.9, 24.0) in the DOR + TAF/FTC arm, −2.2 mg/dL (n = 26; −21.2, 16.8) in the DOR + TDF/FTC arm, and 4.4 mg/dL (n = 23; −15.9, 24.6) in the INSTI + TAF/FTC arm; 1.3 (−10.0, 7.5), −12.4 (21.2, −3.6), and −1.9 mg/dL (−11.5, 7.7), respectively; for LDL; and 2.7 (−2.2, 7.6), −3.4 (−8.2, 1.4), and 2.6 (−2.6, 7.8) mg/dL; respectively, for HDL (analyses excluded those on lipid-lowering therapy). The estimated mean changes in HOMA-IR were 1.77 (n = 32; 95% CI: −1.38, 4.93) for DOR + TAF/FTC, 1.21 (n = 38; −1.69, 4.11) for DOR + TDF/FTC, and −1.00 (n = 40, −3.83, 1.83) for INSTI + TAF/FTC (participants on insulin excluded). Estimated mean treatment differences were not significant for the change in triglycerides, LDL, HDL (Figure 2*A*), or HOMA-IR (Figure 2*B*).

### DEXA Body Composition Change at 48 Weeks

**Figure 3.**
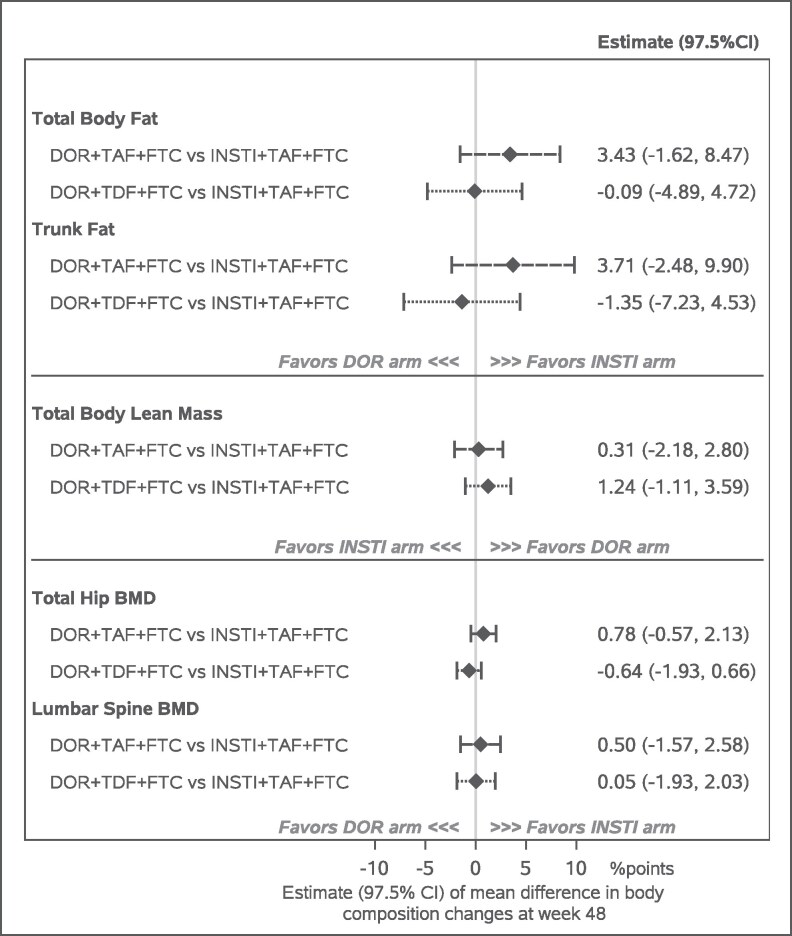
*A*, Estimated mean treatment differences in changes in DEXA total fat mass, trunk fat mass, and total lean mass at 48 weeks for DOR + TAF/FTC and DOR + TDF/FTC versus INSTI + TAF/FTC. *B*, Estimated mean treatment differences in changes in DEXA total hip and lumbar bone mineral density at 48 weeks for DOR + TAF/FTC and DOR + TDF/FTC versus INSTI + TAF/FTC.

The estimated mean change in total fat mass at 48 weeks was 0.60% (n = 35; 95% CI: −2.62, 3.82) for DOR + TAF/FTC, −2.92% (n = 42; −5.86, 0.03) for DOR + TDF/FTC, and −2.83% (n = 40; −5.83, 0.16) for INSTI + TAF/FTC. Estimated mean treatment differences were not significant for the change in total fat, trunk fat, or total lean mass (Figure 3).

### Safety

Reported severe or worse (Grades 3+) adverse events, regardless of causality, were similar across arms (DOR + TAF/FTC, n = 11; DOR + TDF/FTC, n = 12; INSTI + TAF/FTC, n = 15). There were no cases of rash and 2 instances of Grade 2 (moderate) transaminase elevations in the DOR arms. There were 2 Grade 4 events (disseminated herpes zoster and intentional overdose) that were not treatment-related and did not result in a treatment change. There was one instance of virologic failure (VL >200 copies/mL on consecutive measurements) in the INSTI + TAF/FTC arm in a participant unable to obtain standard of care medication.

The median change in calculated CrCl at week 48 was 4.9% for DOR + TAF/FTC (n = 39), 7.4% for DOR + TDF/FTC (n = 45), and 0.4% for INSTI + TAF/FTC (n = 43).

The estimated mean changes in DEXA total hip and lumbar vertebral BMD at 48 weeks were 0.60% (n = 35; 95% CI: −0.25, 1.46) and 0.88% (n = 35; −0.44, 2.20), respectively; for DOR + TAF/FTC, −0.81% (n = 40; −1.61, −0.01) and 0.43% (n = 42; −0.78, 1.64), respectively; for DOR + TDF/FTC, and −0.17% (n = 39; −0.98, 0.63) and 0.38% (n = 40; −0.86, 1.61), respectively, for INSTI + TAF/FTC. Estimated mean treatment differences were not significant for the change in total hip or lumbar BMD (Figure 3). Total hip BMD changed from the osteopenia to osteoporosis range for 1 INSTI + TAF/FTC participant, and lumbar vertebral BMD changed from the osteopenia to osteoporosis range for 1 DOR + TAF/FTC participant.

## DISCUSSION

In this randomized, controlled trial in adults with HIV and obesity on a stable INSTI + TAF/FTC regimen, a switch to DOR, with or without a change from TAF/FTC to TDF/FTC, did not reduce or stabilize weight or improve metabolic health at 48 weeks compared to remaining on an INSTI + TAF/FTC. Women and Black participants, 2 groups at higher risk of weight gain in prior studies, each comprised ≈50% of the study cohort and did not derive significant benefit from a regimen change, nor did those with a history of substantial (>10%) weight gain in the first 3 years of INSTI use. Based on the lower confidence interval limits of weight change within arm, neither DOR switch results in 5% or more weight loss at 48 weeks, providing strong evidence that a change to an NNRTI-based regimen, with or without TDF, is not an effective strategy for weight reduction in individuals with HIV and obesity.

The finding that switching off an INSTI and TAF/FTC does not confer substantial weight benefit is somewhat expected given recent shifts in the consensus view of ART agents and body weight. Early reports of differential weight changes were initially interpreted as evidence of a weight-promoting effect of newer agents (eg, INSTIs and TAF) and assumed older drugs (eg, efavirenz [EFV], zidovudine, and TDF) were weight neutral [8, 9, 18]. This framework has been challenged by more recent clinical trials and reevaluations of older data. The ADVANCE trial reported substantially greater weight gain among treatment-naive individuals randomized to DTG versus EFV (both with TDF/FTC), but a subsequent analysis found that common genetic variations in the EFV-metabolizing CYP2B6 enzyme were a central determinant of weight gain and that EFV may be weight-suppressive in some individuals [9, 19]. Subsequently, the SALSA and TANGO studies showed significantly more weight gain in individuals switching from TDF-containing regimens to DTG/3TC versus those who remained on TDF [19, 20]. In contrast, those switching from TAF-containing regimens experienced no significant change in weight, suggesting that the TDF prodrug formulation of tenofovir was also weight-suppressive. Intriguingly, recent data have not been uniform in this area. In the PASO-DOBLE trial, PWH switching from TDF-containing regimens to DTG/3TC were less likely to gain >5% body weight at 48 weeks compared to those switching to BIC/TAF/FTC (20% vs 41%), suggesting both a weight-suppressive effect of TDF and potentially differential outcomes if the switch is tenofovir-sparing versus TAF inclusive [21]. However, the more recent INSTINCT trial assessed a switch from DTG/3TC to BIC/FTC/TAF and found no appreciable effect on weight at 96 weeks (annualized 0.2% vs 0.3%), suggesting the isolated addition of TAF in the context of sustained viral suppression did not impact body weight [22]. Taken together, these studies reinforce the emerging view that contemporary INSTI + TAF regimens are essentially weight neutral.

Our results align with those of the DEFINE study, another prospective ART switch trial specifically focused on weight and metabolism [23]. DEFINE, which randomized adults with sustained viral suppression on an INSTI + TAF/FTC regimen and ≥10% weight gain in the prior 3 years to switch to darunavir/cobicistat/FTC/TAF or to continue their current ART, found no significant change in weight at 24 weeks. Notably, DEFINE's switch strategy was supported by a combination of retrospective analyses of persons who switched from INSTIs to protease inhibitors and lost weight [24] and hypothesized physiologic mechanisms, including decreased adipocyte lipid storage and increased lipolysis. In contrast, the A5391 strategy was primarily supported by the observation of lower weight gain on NNRTIs after treatment initiation.

All A5391 regimens were well-tolerated, with similar rates of treatment-related adverse events and few treatment-related study discontinuations. The improvements in CrCl among those on DOR were expected as the majority of randomized participants discontinued BIC or DTG, which are associated with increases in serum creatinine related to inhibition of organic cation transporter 2 [25]. BMD changes in both DOR arms were not significant compared to INSTI + TAF/FTC at 48 weeks. Notably, this is one of the few recent studies to assess BMD after the introduction of TDF in PWH with long-term viral suppression, which did not result in significant changes. Finally, this was the first randomized trial to compare virological suppression on DOR-based regimens to standard of care INSTI-based regimens, and the lack of virological failures among DOR recipients compares favorably to INSTI recipients.

The A5391 findings represent an important contribution to the growing literature on the impact of ART on body weight and metabolic health. Strengths of the trial include its prospective, multicenter, randomized design, with a primary outcome of body weight and a range of metabolic, body composition, and safety outcomes, which sets it apart from the many prior retrospective observational cohort studies and secondary analyses. By focusing on patients with obesity, the study enrolled individuals at the highest risk of weight-related comorbid conditions. Enrollment prespecified a goal of ≥50% female and Black participants, 2 often underrepresented groups at higher risk of weight gain on ART. Limitations included a lack of blinding of participants to treatment assignment, although the outcomes were objective, analyses were prespecified, and the data were analyzed in a blinded manner. Recruitment was slower than anticipated, which we attribute to missing weight data (prior to the protocol revision), a hesitancy by some participants and providers to switch from TAF to TDF, and the increasing availability of therapies for weight loss (eg, GLP-1 receptor agonists), which may have influenced the final characteristics of the cohort. The trial was powered to detect a ≥3.5% difference in weight change at week 48, although the clinical relevance of smaller changes is debatable. Participants were on an INSTI + TAF/FTC for a median of 3.4 years, and it is unknown whether an effect on weight would have been possible earlier in the treatment history. Notably, >50% of participants across all arms lost weight, suggesting that behavioral changes during study participation may have occurred. Diet and exercise factors underlying weight loss in all arms cannot be described, as these were reported only at baseline, but it was striking that almost 40% reported some degree of food insecurity. While approximately half of the participants started or changed one of a broad range of medications with potential effects on weight, the distribution of these events was similar across arms. Lastly, although the dropout rate exceeded 10%, preplanned sensitivity analyses demonstrated robustness to missing data.

In summary, switching from an INSTI + TAF/FTC to DOR with either TAF/FTC or TDF/FTC did not result in beneficial weight change or stabilization at 1 year among treatment-experienced PWH with obesity, including among women and Black individuals. The rise in obesity among PWH has been accompanied by an increasing burden of cardiometabolic diseases and other comorbidities, and interventions to reduce body weight and improve metabolic health are needed to improve long-term health outcomes. These findings suggest that weight management in PWH and obesity will require interventions that are more evidence-based and effective than simply modifying ART.

## Supplementary Material

ciag196_Supplementary_Data

## Supplementary Data

Supplementary materials are available at *Clinical Infectious Diseases* online. Consisting of data provided by the authors to benefit the reader, the posted materials are not copyedited and are the sole responsibility of the authors, so questions or comments should be addressed to the corresponding author.
