# Supplementary material for: A 48-Week, Randomized Controlled Trial of Doravirine for Individuals With HIV and Obesity on Integrase Inhibitors and Tenofovir Alafenamide: The Do IT Study (ACTG A5391)
Source: Clin Infect Dis. 2026 Mar 18;83(1):e81–9. doi: 10.1093/cid/ciag196 (PMC13393121; doi:10.1093/cid/ciag196)
Supplement: ciag196_Supplementary_Data [file ciag196_supplementary_data.docx]

**Supplemental Material:** A 48-week, Randomized Controlled Trial of Doravirine for Individuals with HIV and Obesity on Integrase Inhibitors and Tenofovir Alafenamide: The Do IT Study (ACTG A5391)

**Supplemental Figure 1:** CONSORT study diagram.

**
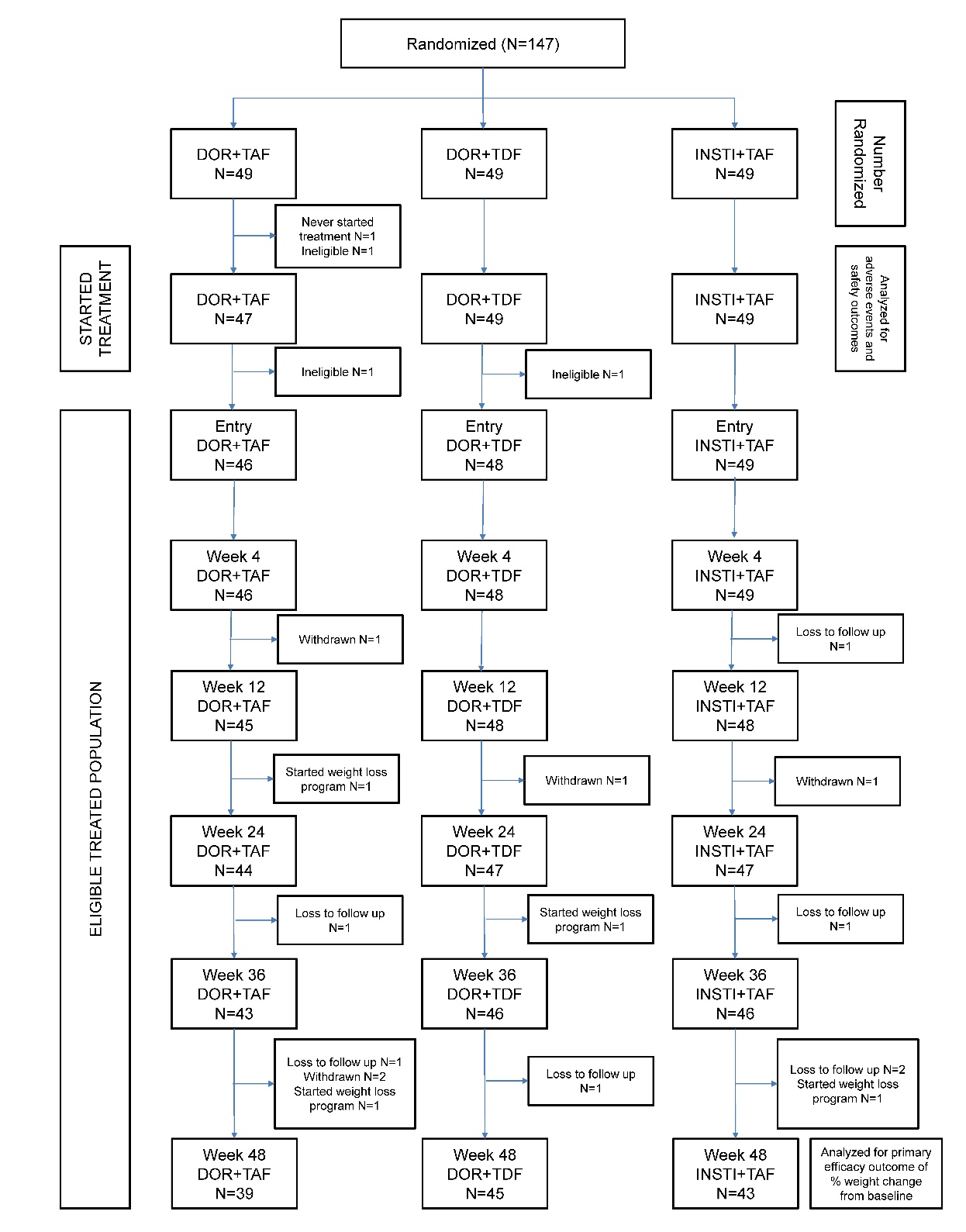
**

The trial randomized 147 participants to three arms; 145 participants initiated the assigned treatment and were included in the safety analysis. Among these, 127 completed 48 weeks on study and comprised the primary efficacy analysis. “Withdrawn” refers to a physician's or participant's decision to leave the study.

**Supplemental Figure 2:** Subgroup estimated treatment differences for **(A)** DOR+TAF/FTC versus INSTI+TAF/FTC and **(B)** DOR+TDF/FTC versus INSTI+TAF/FTC. Race and ethnicity were self-identified by participants. The female gender category included both cisgender women and transgender women receiving feminizing hormone therapy; the “all other” category included all participants not in the female gender category.

**
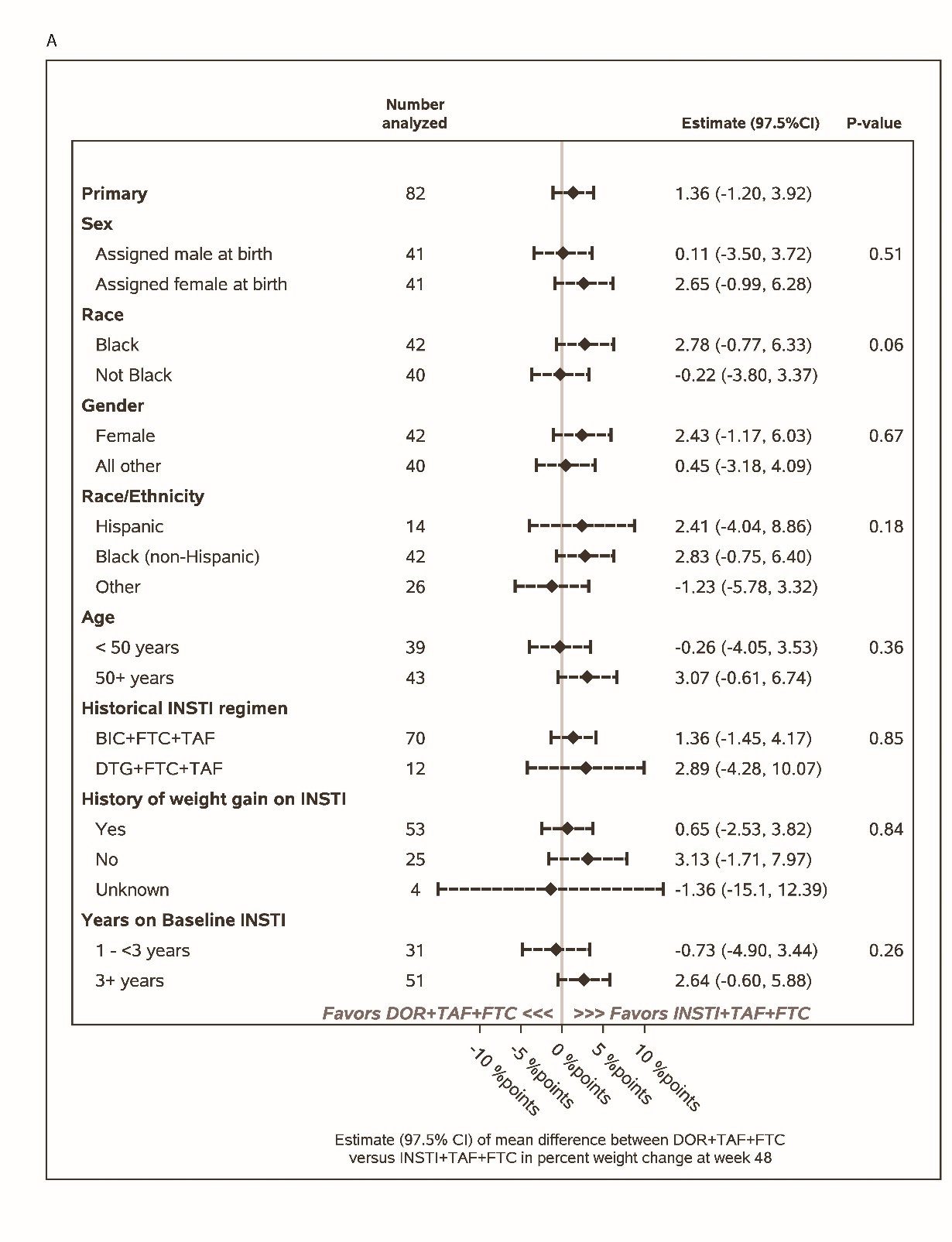
**

**
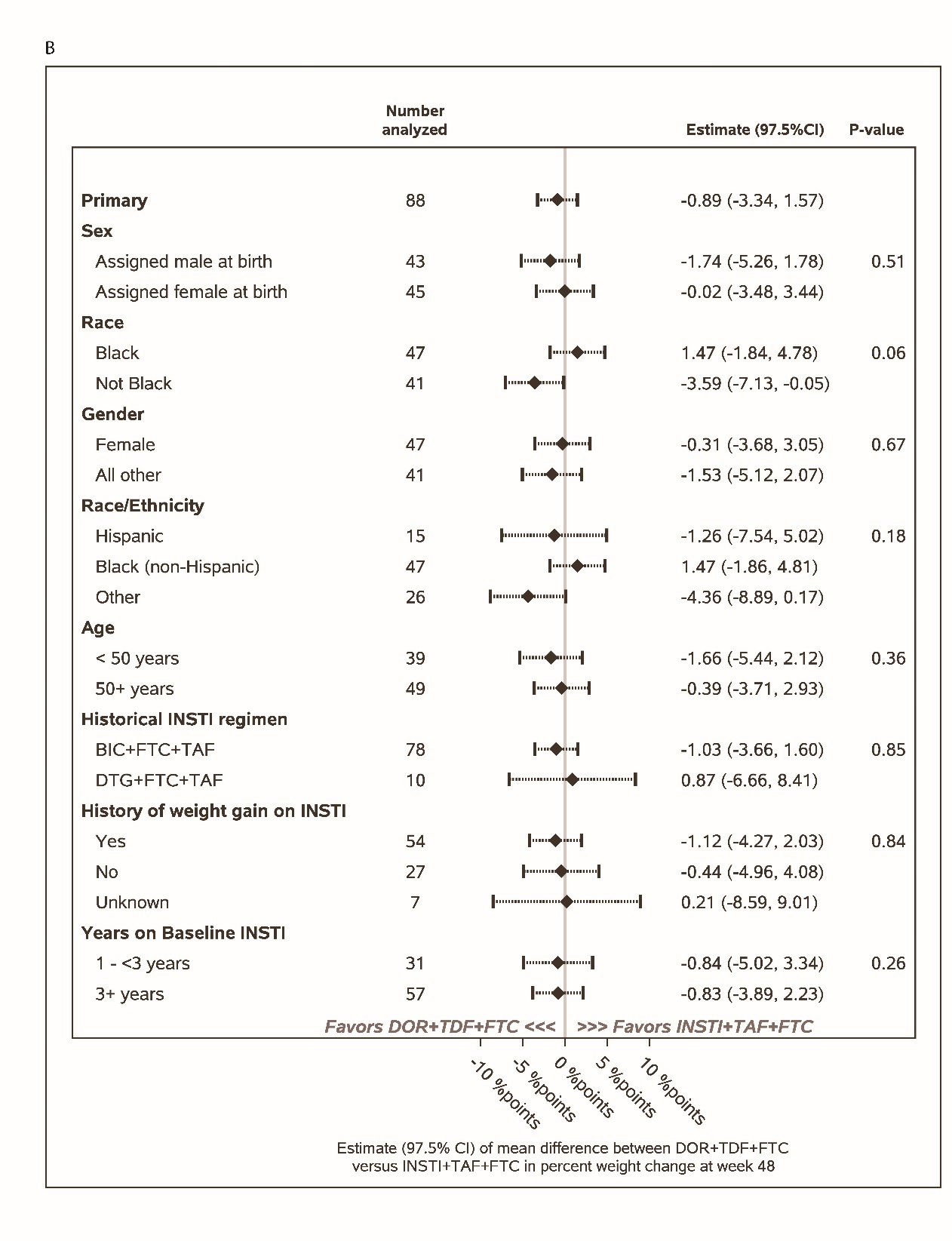
**

**Supplemental** **Figure 3:** Estimated mean treatment differences in changes in waist circumference for DOR+TAF/FTC and DOR+TDF/FTC versus INSTI+TAF/FTC at 48 weeks.


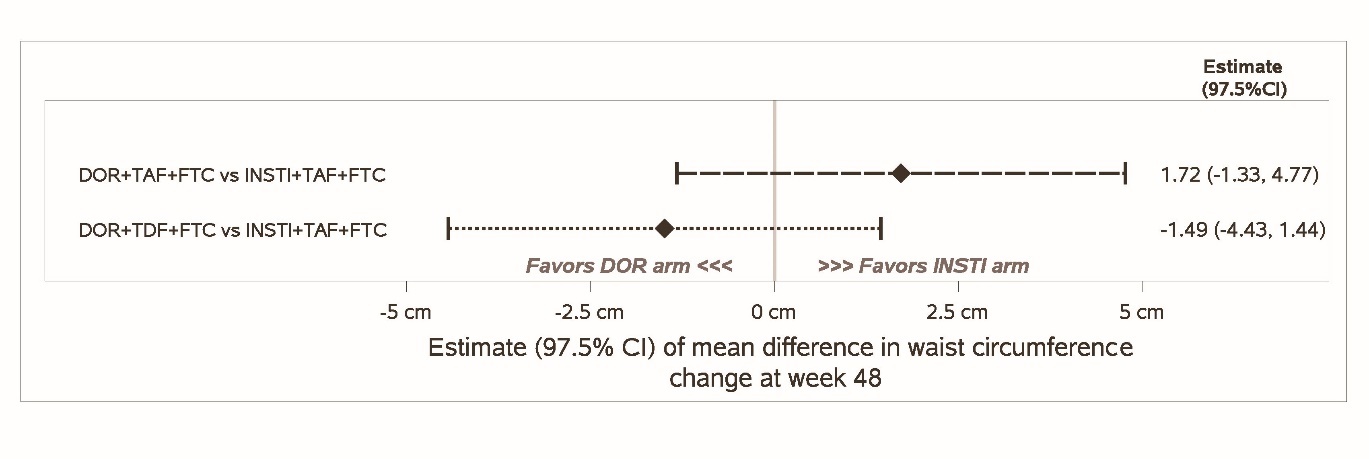


**Supplemental Table:** Medications with a potential impact on weight started or changed by participants during the study.

| **Potential Weight Effect** | **Medication Name** | **Frequency** |
| --- | --- | --- |
| Gain | ALPRAZOLAM | 2 |
|  | AMITRIPTYLINE | 3 |
|  | ARIPIPRAZOLE | 3 |
|  | ATENOLOL | 2 |
|  | CICLOSPORIN | 1 |
|  | CITALOPRAM | 5 |
|  | CITALOPRAM HYDROBROMIDE | 2 |
|  | CLONAZEPAM | 3 |
|  | DEXAMETHASONE | 1 |
|  | DRONABINOL | 1 |
|  | DULOXETINE | 5 |
|  | DULOXETINE HYDROCHLORIDE | 1 |
|  | ESCITALOPRAM | 4 |
|  | ESCITALOPRAM OXALATE | 1 |
|  | FLUOXETINE | 4 |
|  | FLUOXETINE HYDROCHLORIDE | 2 |
|  | GLIMEPIRIDE | 1 |
|  | INSULIN ASPART | 2 |
|  | INSULIN DEGLUDEC | 3 |
|  | INSULIN DETEMIR | 2 |
|  | INSULIN GLARGINE | 2 |
|  | INSULIN LISPRO | 2 |
|  | LITHIUM CARBONATE | 1 |
|  | MEDROXYPROGESTERONE | 1 |
|  | METHADONE | 4 |
|  | METHYLPREDNISOLONE | 5 |
|  | METOPROLOL | 3 |
|  | METOPROLOL SUCCINATE | 8 |
|  | METOPROLOL TARTRATE | 2 |
|  | MINOXIDIL | 1 |
|  | MIRTAZAPINE | 4 |
|  | OLANZAPINE | 2 |
|  | PAROXETINE | 4 |
|  | PREDNISONE | 13 |
|  | PROPRANOLOL | 2 |
|  | QUETIAPINE | 4 |
|  | QUETIAPINE FUMARATE | 7 |
|  | RISPERIDONE | 2 |
|  | SERTRALINE | 7 |
|  | SERTRALINE HYDROCHLORIDE | 1 |
|  | STEROIDS | 1 |
|  | VENLAFAXINE | 3 |
|  | VENLAFAXINE HYDROCHLORIDE | 2 |
| Loss | AMPHETAMINE ASPARTATE; AMPHETAMINE SULFATE; DEXAMPHETAMINE SACCHARATE; DEXAMPHETAMINE SULFATE | 1 |
|  | BACLOFEN | 2 |
|  | BUPROPION | 7 |
|  | BUPROPION HYDROCHLORIDE | 6 |
|  | DULAGLUTIDE | 1 |
|  | EMPAGLIFLOZIN | 1 |
|  | LEVOTHYROXINE | 3 |
|  | LEVOTHYROXINE SODIUM | 2 |
|  | LIRAGLUTIDE | 1 |
|  | LISDEXAMPHETAMINE MESILATE | 1 |
|  | METFORMIN | 9 |
|  | METFORMIN HYDROCHLORIDE | 2 |
|  | NALTREXONE | 2 |
|  | SEMAGLUTIDE | 2 |
|  | TOPIRAMATE | 4 |

*STEROIDS refers to a steroid Injection.*

**A5391 Acknowledgment Appendix:** A5391 trial sites and lead investigators by study enrollment.

Mariano J. Lodigiani, MD and Jesus A. Lara Navas, DDS - Houston Advancing Research Team CRS (Site 31473) Grant UM1AI69432

Rachel Bender Ignacio, MD, MPH and Eli Burnham, PA-C - University of Washington Positive Research CRS (Site 1401) Grant UM1AI69481

Morgan Lima, MSN, RN and Fred Nicotera - Vanderbilt Therapeutics (VT) CRS (Site 3652) Grant UL1TR002243

Cornelius Van Dam, MD and Kelly Phillips, MS, PA-C - Greensboro CRS (Site 3203) Grant UM1AI69423

Sonya L. Heath, MD and Michelle Chambers, MS, CLS, CCRC - Alabama CRS (Site 31788) Grant UM1AI69452

Keisha Ballentine-Cargill, DNP, FNP-BC and Celine Arar, B.A. - Weill Cornell Uptown CRS (Site 7803) Grant UL1 TR002384

Sudipa Sarkar, MD and Patricia Fischer, RN - Johns Hopkins University CRS (Site 201) Grant UM1AI69465

Suzanne Hendler, RN and Carolina Lopez - University of California, San Francisco HIV/AIDS CRS (Site 801) Grant UM1AI69496

Jennifer Manne-Goehler, MD and Hannah Jordan, MPH - Brigham and Women's Hospital Therapeutics (BWH TCRS) CRS (Site 107) Grant UM1AI69412

Carl Fichtenbaum, MD and MaKayla Bishop - Cincinnati CRS (Site 2401) Grant UM1AI69501

Amy Sbrolla RN, BSN, ACRN and Katrina Shea RN, BSN - Massachusetts General Hospital CRS (MGH CRS) (Site 101) Grant UM1AI69412

Penn Therapeutics CRS (Site 6201) Grant UM1AI69534

Vanessa Sutton, NP and Joslyn Axinn, RN - University of Colorado Hospital CRS (Site 6101) Grant UM1 TR004399

Luis Parra Rodriguez, MD and Teresa Spitz, RN - Washington University Therapeutics (WT) CRS (Site 2101) Grant UM1AI069439

Rebecca Fry, FNP and Dan Finn, FNP - Weill Cornell Chelsea CRS (Site 7804) Grant UL1 TR002384

Sarah Henn, MD and Megan Dieterich, PA-C - Whitman-Walker Institute, Inc. CRS (Site 31791) Grant UM1AI154466

Susan Little, MD and Aurora Verduzco Gonzalez, NP - UCSD Antiviral Research Center CRS (Site 701) Grant James B. Pendleton Charitable Trust, P30AI036214

George A. Yendewa, MD, MPH and Jeffrey M. Jacobson, MD - Case CRS (Site 2501) Grant UM1AI69501

Sadia Shaik and Eric Daar - Harbor University of California Los Angeles Center CRS (Site 603) Grant UM1-AI069424, UL1TR000124

Diana Finkel, DO and Zoraida Cruz-Barahona - New Jersey Medical School Clinical Research Center CRS (Site 31786) Grant UM1AI69419

Claudia Hawkins, MD, MPH and Sherryl Wolfe, BSN, RN - Northwestern University CRS (Site 2701) Grant UM1AI69471

Yesha Patel, MD and Jessica Marburger, RN - Ohio State University CRS (Site 2301) Grant UM1AI69494

Maricela Gonzalez and Lisa Mark, PA-C - University of California, Los Angeles CARE Center CRS (Site 601) Grant P30 AI152501; The James B. Pendleton Charitable Trust

Sonal Munsiff, MD and Susan Hulse, PA-C - University of Rochester Adult HIV Therapeutic Strategies Network CRS (Site 31787) Grant UM1AI69511

Jonathan Oakes and Deborah Pettus - Chapel Hill CRS (Site 3201) Grant UL1TR002489, UM1AI69423

Ariana Pazmino, MS, RN and Brett Gray, ANP, MPH - Columbia Physicians & Surgeons (P&S) CRS (Site 30329) Grant UM1AI69470

Ericka R. Patrick, RN, MSN and Melody Palmore, MD - The Ponce de Leon Center CRS (Site 5802) Grant P30AI050409, UM1AI69418
